# Chest compression during sustained inflation versus 3:1 compression-to-ventilation ratio during neonatal cardiopulmonary resuscitation of asphyxiated piglets

**DOI:** 10.1016/j.resplu.2025.101144

**Published:** 2025-10-28

**Authors:** Melanie Shaker, Anne Lee Solevåg, Megan O’Reilly, Georg M. Schmölzer

**Affiliations:** a Centre for the Studies of Asphyxia and Resuscitation, Neonatal Research Unit, Royal Alexandra Hospital, Edmonton, Alberta, Canada; b Department of Neonatal Intensive Care, Division of Paediatric and Adolescent Medicine, Oslo University Hospital, Rikshospitalet, Oslo, Norway; c Institute of Clinical Medicine, Faculty of Medicine, University of Oslo, Oslo, Norway; d Department of Pediatrics, University of Alberta, Edmonton, Alberta, Canada

**Keywords:** Infants, Newborn, Neonatal resuscitation, Chest compressions, Asphyxia, Sustained inflation

## Abstract

**Background:**

Current neonatal resuscitation guidelines recommend using the 3:1 chest compression-to-ventilation (C:V) ratio technique. However, an alternative technique using continuous compressions superimposed with a high distending pressure or sustained inflation (CC + SI) may improve return of spontaneous circulation (ROSC), survival, and post-resuscitation outcomes.

**Objective:**

In a piglet model of asphyxia-induced cardiac arrest, compare time to ROSC with CC + SI or 3:1C:V technique for providing neonatal cardiopulmonary resuscitation (CPR).

**Methods:**

Secondary analysis of 132 term newborn mixed breed piglets (1–3 days of age, weighing 1.7–2.4 kg) from six different studies, which were exposed to 30–50 min of normocapnic hypoxia followed by asphyxia until cardiac arrest. This was followed by CPR with either the CC + SI or 3:1C:V technique.

**Results:**

Although the proportion of piglets achieving ROSC was similar between CC + SI and 3:1C:V [59/83 (71 %) vs. 40/49 (82 %)], the time to ROSC was significantly shorter with CC + SI [median (IQR), 87.5 (66.8–147.5) vs. 120 (76.5–267) s; p = 0.0097], corresponding to a mean difference of –73.9 s (95 % CI –122.5 to –25.3). Survival up to 4 h did not differ between groups (risk ratios 1.04, 95 % Confidence intervals 0.82–1.32), with mean (SD) survival time among ROSC survivors of 237 (18) min for CC + SI vs 220 (55) min for 3:1C:V (p = 0.0623). In adjusted analyses, CC + SI yielded faster time to ROSC (Geometric Mean Ratio 0.67, 95 % CI 0.50–0.88), with no effect modification by FiO_2_, but a rate-dependent effect on time to ROSC; 4-h survival did not differ between methods.

**Conclusions:**

Use of the CC + SI technique during neonatal piglet resuscitation leads to a faster ROSC, with no difference in survival.

## Introduction

The need for cardiopulmonary resuscitation (CPR) at birth occurs in approximately 10–15 % of preterm infants and 0.1 % of term infants worldwide.^1^, ^2^, ^3^ Infants that receive CPR at birth face a substantially high mortality, with up to half not surviving resuscitation. A multitude of survivors experience significant neurological morbidities: 57 % encounter hypoxic-ischemic encephalopathy (brain damage) and seizures, and 73 % suffer from long-term neurological disabilities.^4^, ^5^, ^6^, ^7^, ^8^ The poor prognosis associated with neonatal CPR raises the question as to whether a change in current resuscitation techniques could improve survival and neurological outcomes.

The current standard of care for newborns requiring CPR is a coordinated 3:1compression-to-ventilation (C:V) ratio.^1^, ^2^, ^3^ This is composed of 90 chest compressions (CC) and 30 ventilations per minute, with a pause after every third CC to deliver one ventilation.^1^, ^2^, ^3^ However, animal studies comparing 3:1C:V with other C:V ratios (i.e., 2:1, 9:3, 15:2) did not report any difference in time to return of spontaneous circulation (ROSC), rate of RSOC or survival to the end of experiment.^9^, ^10^, ^11^ Similarly, when 3:1C:V was compared to continuous chest compression with asynchronous ventilation in animal models, no difference in time to ROSC or survival to the end of experiment was reported.^12^, ^13^, ^14^ These findings suggest that neither changing the C:V ratio nor continuous chest compression with asynchronous ventilation improves outcomes.

An alternative approach might be chest compressions combined with sustained inflation (CC + SI), which delivers a constant high distending pressure during continuous chest compression. CC + SI provides passive ventilation (= lung inflation during chest recoil) and thereby achieves lung recruitment compared to 3:1C:V or continuous chest compression with asynchronous ventilation, which has been shown to cause lung derecruitment.^15^ Single animal and human studies reported that CC + SI reduces time to ROSC compared to 3:1C:V.^16^, ^17^, ^18^, ^19^, ^20^, ^21^, ^22^, ^23^, ^24^ Although individual studies have demonstrated that CC + SI shortens time to ROSC, these findings have not been systematically validated across multiple experiments or linked to survival outcomes; therefore, we combined data from several studies to determine whether the reduction in time to ROSC with CC + SI is accompanied by improved survival. We hypothesized that using CC + SI would have a shorter time to ROSC and higher survival to the end of experiment compared to the 3:1C:V technique.

## Methods

**Fig. 1:**
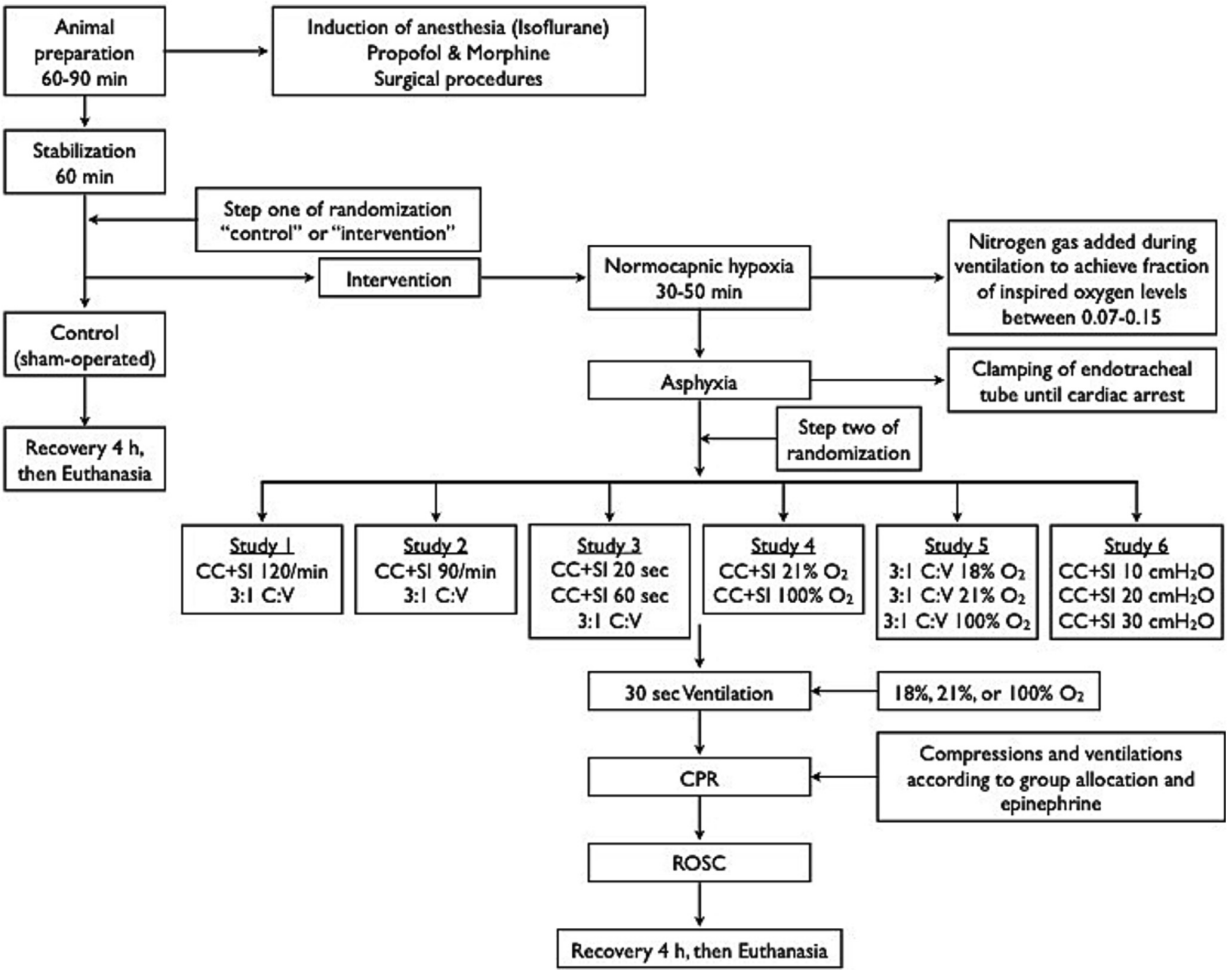
Study flow chart.

We performed a database analysis of six previous experiments from our laboratory.^16^, ^17^, ^18^, ^19^, ^20^, ^21^ A total of 132 term newborn mixed breed duroc piglets (1–3 days of age, weighing 1.7–2.4 kg) were obtained on the day of experimentation from the University Swine Research Technology Centre. All experiments were conducted in accordance with the guidelines and approval of the Animal Care and Use Committee (Health Sciences), University of Alberta (AUP00001764, AUP00002151, AUP00002651), and were conducted according to either the ARRIVE or ARRIVE 2.0 guidelines.^25^, ^26^ The study protocol is presented in Fig. 1 and the SYCRLE risk of bias assessment is presented.^27^ The SYRCLE risk of bias tool was developed specifically for the appraisal of preclinical animal studies. Unlike the Cochrane risk of bias tool, which was designed for clinical trials, the SYRCLE tool adapts the same methodological principles to address sources of bias more relevant to animal research, such as allocation concealment, random housing, blinding of caregivers, and random outcome assessment.^27^

*Animal preparation* is detailed in each of the publications.^16^, ^17^, ^18^, ^19^, ^20^, ^21^ In short, all piglets were intubated via a tracheostomy and ventilated using pressure-controlled ventilation (Acutronic Fabian HFO; Hirzel, Switzerland), while maintaining oxygen saturation, glucose level, hydration, and body temperature. *Anaesthesia* was maintained with intravenous propofol and morphine. A 5-French Argyle® catheter (Klein-Baker Medical Inc. San Antonio, TX) was inserted above the right renal artery via the femoral artery for *continuous arterial blood pressure monitoring* in addition to arterial blood gas measurements. The right common carotid artery was also exposed and encircled with a real-time ultrasonic flow probe (2 mm; Transonic Systems Inc., Ithica, NY) to measure *cerebral blood flow*. A 5-French Argyle® catheter (Klein-Baker Medical Inc. San Antonio, TX) was inserted just before the right atrium via the femoral vein for *continuous central venous pressure monitoring*.

A respiratory function monitor (NM3, Respironics, Philips, Andover, MA) was used to continuously measure *inspired and expired tidal volume, airway pressures, gas flow, and end-tidal CO_2_*.^28^, ^29^ *Cerebral oxygenation* was measured using the Invos^TM^ Cerebral/Somatic Oximeter Monitor (Invos 5100, Somanetics Corp., Troy, MI).^30^

### Experimental protocols for included studies

*For all studies:* All studies included a sham-operated control group, which received the same surgical protocol, stabilization, and equivalent experimental periods without hypoxia and asphyxia. To reduce the occurrence of selection bias, a two-step randomization process was used. Following surgical instrumentation and the stabilization procedure, a subsequently numbered, sealed opaque envelope containing the assignment “sham” or “intervention” was opened (step one). Piglets that were randomized to “intervention” underwent both hypoxia and asphyxia, whereas the piglets randomized to “sham” were not. Upon meeting the criteria for resuscitation, a second sequentially numbered, sealed opaque envelope, containing the intervention assignment for each study group (step two) was opened. The piglets that were randomized to “intervention” were exposed to normocapnic hypoxia, which was followed by asphyxia, which was achieved by being disconnected from the ventilator and clamping of the endotracheal tube until cardiac arrest. Fifteen seconds after cardiac arrest, positive pressure ventilation (PPV) was commenced for 30 s with a Neopuff T-Piece (Fisher & Paykel, Auckland, New Zealand). Unless specified by the intervention group allocation, the default settings were a peak inflating pressure of 30 cmH_2_O, a positive end expiratory pressure of 5 cmH_2_O, and a gas flow of 8 L/min. Chest compressions (CCs) were performed using the two-thumb encircling technique in all piglets. A metronome was used to achieve the targeted compression rate. After 30 s of CCs, 100 % oxygen was commenced (unless piglets were randomized to receive 18 % or 21 % oxygen). Epinephrine (0.01–0.02 mg/kg per dose) was administered at 2, 5, and 8 min after start of CPR. CPR was performed for a maximum of 10 min. ROSC was defined as an unassisted heart rate >100/min. After ROSC, piglets recovered for four hours before being euthanized with an intravenous overdose of sodium pentobarbital (120 mg/kg).

*Study interventions:* Piglets were randomized to receive CPR using the CC + SI technique received continuous CCs at a rate of 90 or 120/min (according to study protocol). SI was delivered during CCs with a peak inflating pressure of 10, 20, or 30 cmH_2_O (according to study protocol) for a duration of 20, 30, or 60 s (according to study protocol). SI was interrupted for 1 s before a further SI was provided. CPR in the 3:1C:V group was performed with a CC rate of 90/min and 30 inflations, delivered at a ratio of 3:1compressions-to-ventilations, as per current resuscitation guidelines.^1^, ^2^, ^31^, ^32^

*Study 1*: Piglets were randomized to receive CC + SI delivered at a compression rate of 120/min, or 3:1C:V.^16^ *Study 2*: Piglets were randomized to receive CC + SI delivered at a compression rate of 90/min, or 3:1C:V.^17^ *Study 3*: Piglets were randomized to receive CC + SI with SI duration inflation of 20 s or 60 s, or 3:1C:V.^18^ *Study 4*: Piglets were randomized to receive 21 % or 100 % oxygen during CC + SI.^21^ *Study 5*: Piglets were randomized to receive 18 %, 21 %, or 100 % oxygen during 3:1C:V.^20^ *Study 6*: Piglets were randomized to receive CC + SI with the SI delivered at a pressure of 10 cmH_2_O, 20 cmH_2_O, or 30 cmH_2_O.^19^

### Data collection and analysis

Transonic flow probes, heart rate and pressure transducer outputs were digitized and recorded with LabChart® programming software (ADInstruments, Houston, TX). Only tissue samples from surviving piglets four hours after intervention were collected at post-mortem. Lung tissue from the right basal part of the lung lobe was snap frozen in liquid nitrogen before being stored at –80 °C; the brain was removed from the skull and placed in ice-cold 2-methylbutane for 10 min before being stored at –80 °C. Lung and brain tissue samples were homogenized in a lysis buffer (0.5 % Tween-20/PBS containing a protease inhibitor cocktail). Homogenized samples were centrifuged at 3000 × g for 10 min at 4 °C. The supernatants were retained, and protein concentration was quantified using the Bradford method. Evidence of lung and brain injury was determined by quantification of the concentrations of the pro-inflammatory cytokines interleukin (IL)-1β, -6, -8, and tumor necrosis factor (TNF)-α in lung and brain tissue homogenates using commercially available ELISA kits (PLB00B, P6000B, P8000, PTA00, R&D Systems, Minneapolis, USA). Cytokine concentrations were quantified according to protocols provided by the manufacturer and were expressed relative to protein concentration.

### Statistical analysis

Data are presented as mean (standard deviation, SD) for normally distributed continuous variables and as median (interquartile range, IQR) when distributions was skewed. For all respiratory parameters, continuous values during resuscitation were analyzed. We defined the volume going into and out of the lung as follow: Any air entering the lung during either inflation or chest compression was defined as inspired tidal volume, and any air leaving the lung during chest compression was defined as expired tidal volume.^15^ Normality was assessed, and comparisons were performed using Student’s *t*-test for parametric and Mann-Whitney *U* test for non-parametric comparisons of continuous variables, and χ^2^ for categorical variables. Furthermore, we analysed these three complementary outcomes, proportion with ROSC, time to ROSC (animals how achieved ROSC), and total CPR time (all animal regardless of achieving ROSC or not). All effect estimates are presented with 95 % confidence intervals (CIs). For binary outcomes, risk ratios (RRs) and risk differences (RDs) with 95 % CIs were calculated; for continuous outcomes, mean differences (MDs) with 95 % CIs were reported. We fit multivariable models for ROSC (logistic), 4-h survival (logistic), and log-transformed time to ROSC (linear), adjusted for age, weight, FiO_2_, and compression rate. To assess effect modification, we included method × FiO_2_ and method × rate interaction terms. Because sustained-inflation pressure and duration are only applicable to CC + SI, we modelled them as CC + SI-only interaction terms (method × pressure, method × duration). Main method effects are reported from adjusted models without interactions; interaction p-values are from models including the interaction terms. We modelled log(time to ROSC) and reported geometric mean ratios (GMR = exp[β]) with 95 % CIs; values < 1 indicate shorter times with CC + SI. A Kaplan-Meier survival curve was generated to compare outcomes between CC + SI and 3:1C:V techniques. *P*-values are 2-sided and *p* < 0.05 was considered statistically significant. Statistical analyses were performed with SigmaPlot (Systat Software Inc., San Jose, CA) and SAS Ver. 9.4 (SAS Institute Inc., Cary, NC).

## Results

Newborn mixed breed piglets (n = 132) were obtained on the day of the experiment and were subjected to the hypoxia-asphyxia protocol of the specific study. There was no significant difference in the age or weight of piglets in the CC + SI group [2.0 (0.9) days, 2.0 (0.2) kg] compared to the 3:1C:V group [2.1 (1.0) days, 2.0 (0.2) kg]. Baseline demographics, hemodynamics and blood gas parameters were similar between CC + SI and 3:1C:V groups (Tables 1).

**Table 1:** Demographics and baseline blood gas values and hemodynamic parameters.

	**CC + SI**	**3:1C:V**	**p-value**
Weight (kg)	2 (0.19)	2 (0.19)	0.415
Age (days)	2 (0.9)	2.1 (0.95)	0.401
Male*	55 (66)	29 (59)	0.457
Heart rate (beats/min)	203 (34)	200 (39)	0.692
MAP (mmHg)	62 (13)	62 (9)	0.765
Systolic BP (mmHg)	80 (16)	81 (13)	0.617
Diastolic BP (mmHg)	31 (12)	35 (11)	0.175
CVP (mmHg)	5 (2)	5 (1)	0.554
Carotid flow (mL/min)	50 (20)	50 (16)	0.984
Cerebral oxygenation (%)	53 (15)	52 (13)	0.783
pH	7.47 (0.09)	7.45 (0.08)	0.443
PaO_2_ (mmHg)	87.0 (20.6)	85.3 (18.8)	0.637
PaCO_2_ (mmHg)	35 (6)	35 (5)	0.642
Hemoglobin (g/dL)	7.5 (1.2)	7.8 (1.0)	0.164
Base excess (mmol/L)	1.0 (3.9)	0.6 (3.6)	0.518
HCO_3_^−^ (mmol/L)	24.9 (2.9)	24.6 (2.7)	0.630
Lactate (mmol/L)	3.9 (1.2)	3.7 (0.9)	0.407

**Fig. 2:**
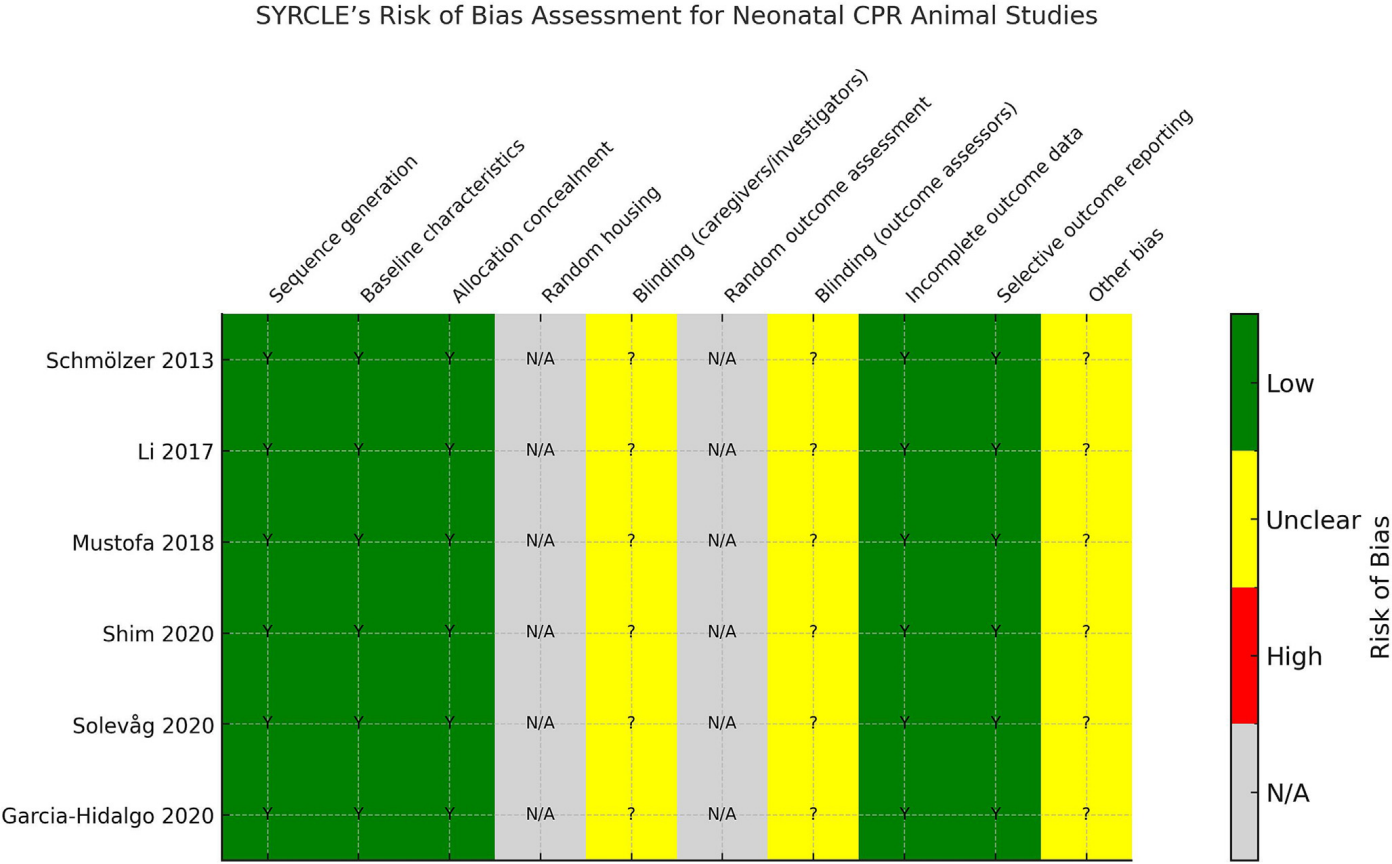
SYRCLE’s risk of bias assessment.

Risk of bias across the six included animal studies was assessed using the SYRCLE Risk of Bias tool. All studies demonstrated low risk for sequence generation, baseline characteristics, and allocation concealment (Fig. 2). Domains such as random housing and random outcome assessment were deemed not applicable, given the tightly controlled experimental laboratory conditions. The domain assessing blinding of outcome assessors was rated as unclear risk due to insufficient detail in reporting blinding procedures. Caregiver/investigator blinding was also rated as unclear, as blinding during real-time CPR interventions was not possible. Most studies showed low risk in handling incomplete data and selective outcome reporting. Overall, internal validity was rated favorably, although some domains remained vulnerable to reporting bias due to methodological limitations inherent in preclinical experimental design.

### Resuscitation

Duration of asphyxia (Table 2) and the degree of asphyxiation (as indicated by pH, PaCO_2_, base excess, and lactate; Table 4) were similar between CC + SI and 3:1C:V groups. A total of 63/83 (76 %) CC + SI and 43/49 (88 %) 3:1C:V piglets achieved ROSC (Table 2). The time to ROSC was significantly shorter with CC + SI [median (IQR), 87.5 (66.8–147.5) vs. 120 (76.5–267) s; p = 0.0097], corresponding to a mean difference of –73.9 s (95 % CI –122.5 to –25.3) (Table 2). The CPR Time (all cases) was not different between groups with CC + SI 120.0 (75.5–372.0)sec vs. 188.0 (80.0–349.0)s with 3:1C:V p = 0.449.

**Table 2:** Characteristics of asphyxia, resuscitation, and survival of asphyxiated piglets.

	**CC + SI (n = 83)**	**3:1C:V (n = 49)**	**p-value**
Asphyxia time (sec)	354 (170)	359 (180)	0.88
Epinephrine doses (n)	1.3 (1.7)	1.4 (1.6)	0.746
Achieving ROSC^†^	63 (76 %)	43 (88 %)	0.176
ROSC time (sec) ^#^	90 (69–180)	160 (77–300)	0.0097
Survival 4 h after ROSC^†^	60 (95 %)	40 (93 %)	0.176
Survival time after ROSC (min)	237 (18)	220 (55)	0.0623

**Fig. 3:**
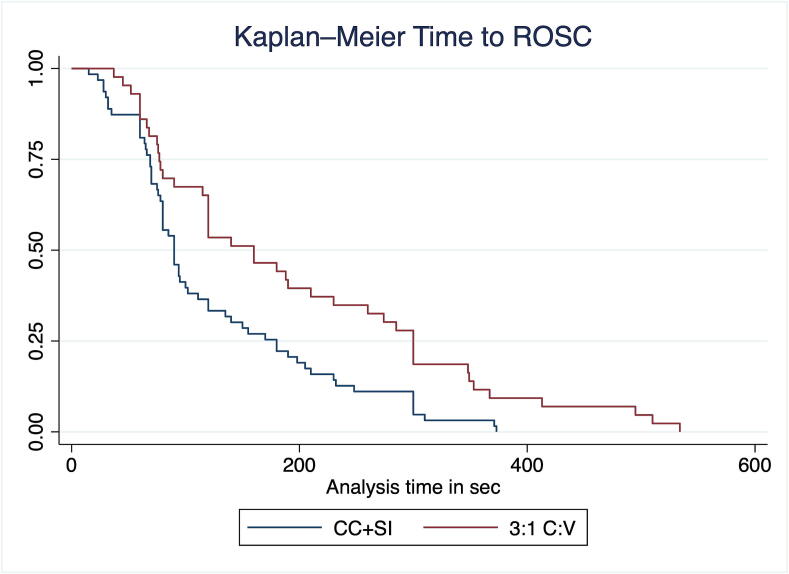
Kaplan-Meier Time to achieve return of spontaneous circulation (ROSC) curve.

Data are presented as mean (SD), unless indicated ^†^n (%), ^#^median (IQR), 3:1C:V = 3:1compression-to-ventilation ratio, CC + SI = continuous compressions superimposed with sustained inflation, ROSC = return of spontaneous circulation,

Piglets in the CC + SI group had improved time to ROSC compared to the 3:1C:V group, as indicated in the Kaplan-Meier survival curve (Fig. 3). Survival up to 4 h did not differ between groups (RR 1.04, 95 % CI 0.82–1.32), with mean (SD) survival time among ROSC survivors of 237 (18) min for CC + SI vs 220 (55) min for 3:1C:V (p = 0.0623).

Adjusted analyses confirmed faster time to ROSC with CC + SI (GMR 0.67, 95 % CI 0.50–0.88; p = 0.005). FiO_2_ did not modify the effect (interaction p = 0.11), whereas a significant method × rate interaction was present for time to ROSC (interaction p < 10^-36^); pressure and duration (CC + SI-only) were not effect modifiers (interaction p ≥ 0.27). Adjusted odds of ROSC were lower with CC + SI (Odds Ratio 0.30, 95 % CI 0.10–0.91), and 4-h survival was similar (Odds Ratio 0.93, 95 % CI 0.38–2.23).

### Changes in hemodynamic and blood gas parameters

**Fig. 4:**
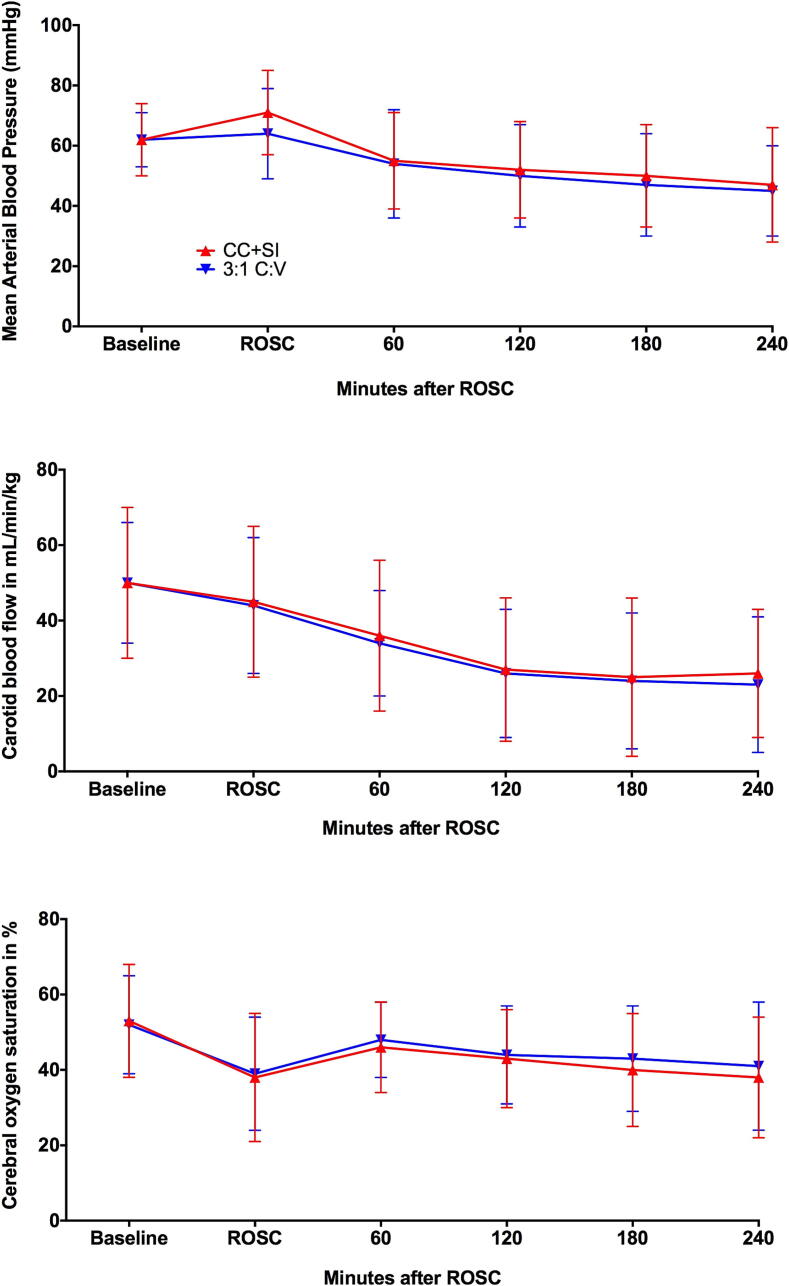
Changes in mean arterial blood pressure (mmHg), carotid blood flow (mL/min/kg), and cerebral oxygen saturation (%) during baseline, ROSC, and post-resuscitation (minutes after ROSC).

Compared to baseline, the heart rate increased after resuscitation and ROSC in both CC + SI and 3:1C:V piglets and remained higher than baseline values for the duration of the 4-h post-resuscitation recovery period (Table 3). At 10 min after ROSC, piglets resuscitated with CC + SI presented with a significantly higher mean (SD) mean arterial blood pressure (MAP) compared to 3:1C:V piglets [71 (15) mmHg vs. 64 (15) mmHg, p = 0.020], however MAP remained similar between groups and lower than baseline values for the remainder of the 4-h post-resuscitation recovery period (Table 3, Fig. 4). Carotid artery blood flow and cerebral oxygenation also decreased from baseline level throughout the 4-h recovery period and were similar between groups (Table 3). Table 4 shows the blood gas parameters (pH, PaO_2_, PaCO_2_, hemoglobin, base excess, HCO_3_^−^, and lactate) at baseline, after asphyxia, and at 1-h and 4-h post-resuscitation in CC + SI and 3:1C:V groups.

**Table 3:** Hemodynamics after resuscitation.

		**CC + SI**	**3:1C:V**
**Heart rate (beats/min)**			
After ROSC	10 min	248 (31)	251 (30)
	1 h	251 (48)	254 (54)
	2 h	281 (270)	245 (56)
	3 h	258 (50)	251 (42)
	4 h	237 (49)	236 (40)

**MAP (mmHg)**			
After ROSC	10 min	71 (15)	64 (15)
	1 h	55 (16)	54 (18)
	2 h	52 (16)	50 (17)
	3 h	50 (17)	47 (17)
	4 h	47 (20)	45 (15)

**Systolic BP (mmHg)**			
After ROSC	10 min	87 (19)	84 (20)
	1 h	68 (15)	72 (21)
	2 h	62 (16)	68 (20)
	3 h	60 (16)	66 (17)
	4 h	59 (19)	66 (21)

**Diastolic BP (mmHg)**			
After ROSC	10 min	51 (12)	46 (11)
	1 h	39 (10)	41 (16)
	2 h	37 (11)	41 (15)
	3 h	35 (11)	37 (10)
	4 h	31 (12)	35 (11)

**CVP (mmHg)**			
After ROSC	10 min	6 (2)	7 (6)
	1 h	5 (2)	5 (2)
	2 h	4 (2)	5 (2)
	3 h	4 (2)	4 (2)
	4 h	5 (2)	5 (2)

**Carotid flow (mL/min)**			
After ROSC	10 min	45 (20)	44 (18)
	1 h	36 (20)	34 (14)
	2 h	27 (19)	26 (17)
	3 h	25 (22)	24 (18)
	4 h	26 (17)	23 (18)

**Cerebral oxygenation (%)**			
After ROSC	10 min	56 (11)	56 (13)
	1 h	46 (12)	48 (10)
	2 h	43 (13)	44 (13)
	3 h	40 (15)	43 (14)
	4 h	38 (16)	41 (17)

Data are presented as mean (SD), 3:1C:V = 3:1compression-to-ventilation ratio, CC + SI = continuous compressions superimposed with sustained inflation, ROSC = return of spontaneous circulation, MAP = mean arterial blood pressure, BP = blood pressure, CVP = central venous pressure.

**Table 4:** Blood gases after resuscitation.

		**CC + SI**	**3:1C:V**
**pH**			
After asphyxia		6.58 (0.09)	6.62 (0.14)
After ROSC	1 h	7.21 (0.13)	7.18 (0.17)
	4 h	7.28 (0.16)	7.31 (0.14)

**PaO_2_ (mmHg)**			
After ROSC	1 h	103.1 (30.0)	102.0 (23.7)
	4 h	82.5 (26.3)	86.8 (21.0)

**PaCO_2_ (mmHg)**			
After asphyxia		94 (83–102)	92 (81–104)
After ROSC	1 h	33.3 (10.5)	35.0 (9.8)
	4 h	37.7 (9.2)	38.3 (8.4)

**Hemoglobin (g/dL)**			
After asphyxia		8.0 (1.3)	8.2 (1.2)
After ROSC	1 h	8.3 (1.4)	8.4 (1.1)
	4 h	8.4 (1.7)	8.4 (1.4)

**Base excess (mmol/L)**			
After asphyxia		−26.9 (2.5)	−25.8 (2.5)
After ROSC	1 h	−15.0 (4.6)	−15.0 (5.0)
	4 h	−8.5 (7.1)	−9.6 (16.6)

**HCO_3_^−^ (mmol/L)**			
After asphyxia		10.2 (2.0)	10.7 (1.8)
After ROSC	1 h	14.0 (5.8)	13.4 (3.0)
	4 h	18.2 (4.8)	22.2 (15.7)

**Lactate (mmol/L)**			
After asphyxia		15.6 (2.3)	15.1 (1.7)
After ROSC	1 h	12.5 (3.1)	12.9 (2.9)
	4 h	6.4 (4.6)	5.5 (3.1)

Data are presented as mean (SD), 3:1C:V = 3:1compression-to-ventilation ratio, CC + SI = continuous compressions superimposed with sustained inflation, ROSC = return of spontaneous circulation, PaO_2_ = partial pressure of oxygen in arterial blood, PaCO_2_ = partial pressure of carbon dioxide in arterial blood, HCO_3_^−^ = bicarbonate ion

### Respiratory parameters

Respiratory parameters during resuscitation are presented in Table 5. The delivered tidal volume during 3:1C:V was significantly higher compared to CC + SI, and the minute ventilation was significantly lower in 3:1C:V compared to CC + SI.

**Table 5:** Respiratory parameters during resuscitation.

	**CC + SI**	**3:1C:V**	**p-value**
Tidal volume (mL/kg)	14.1 (3.8)	19.2 (3.5)	<0.0001
Minute ventilation (mL/kg/min)	964 (201)	523 (116)	<0.0001
Peak inflation pressure (cmH_2_O)	43.2 (5.3)	29.4 (0.6)	<0.0001

Data are presented as mean (SD) 3:1C:V = 3:1compression-to-ventilation ratio, CC + SI = continuous compressions superimposed with sustained inflation

### Injury markers

**Fig. 5:**
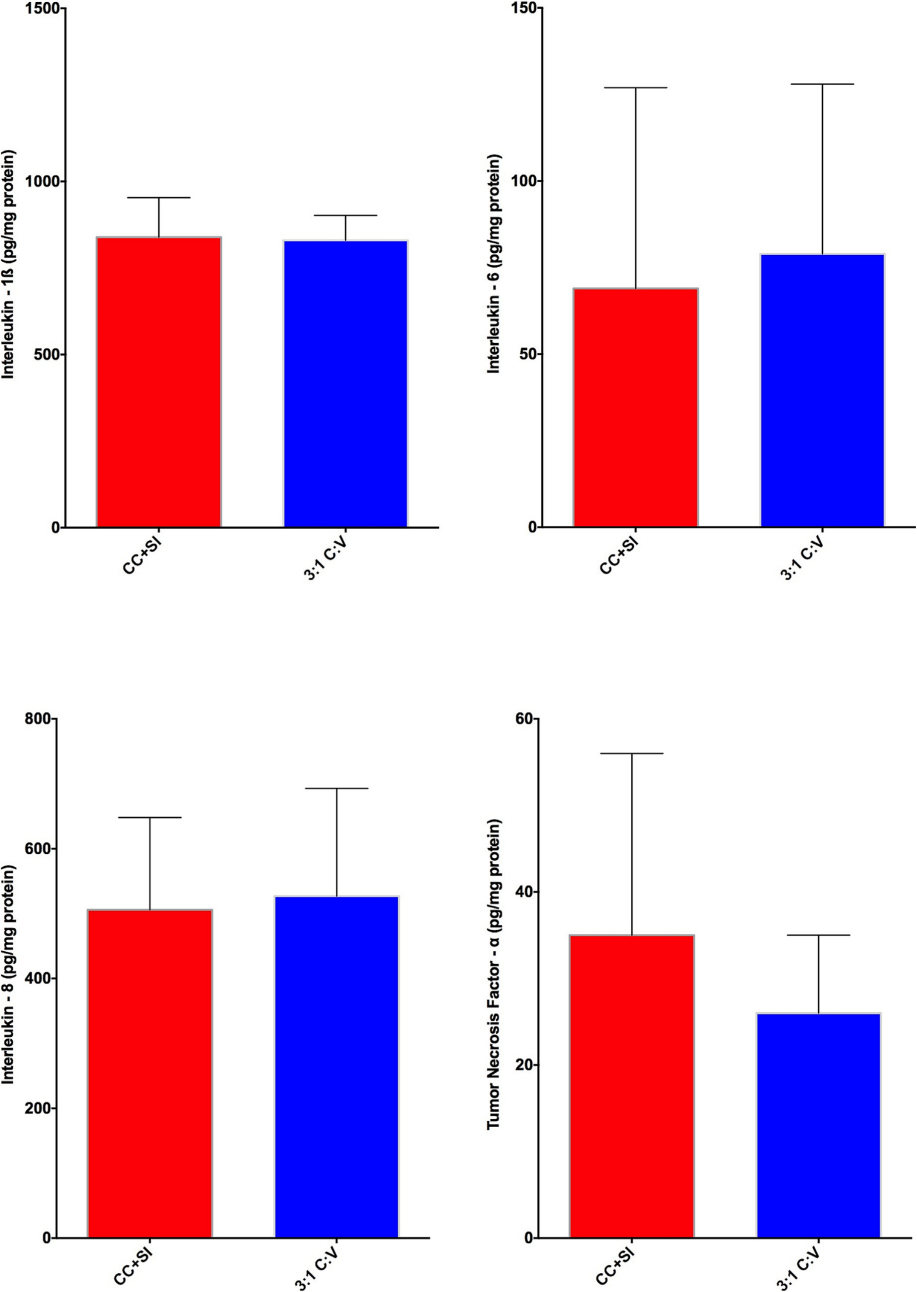
Concentrations of TNF-α, IL-1β, IL-6, and IL-8 in lung tissue homogenates, expressed relative to lung protein concentration in piglets resuscitated using chest compressions during sustained inflations (CC + SI) or 3:1compression:ventilation ratio (3:1C:V). Results represent the median (solid bar), IQR (box margin), and 95 % confidence interval.

**Fig. 6:**
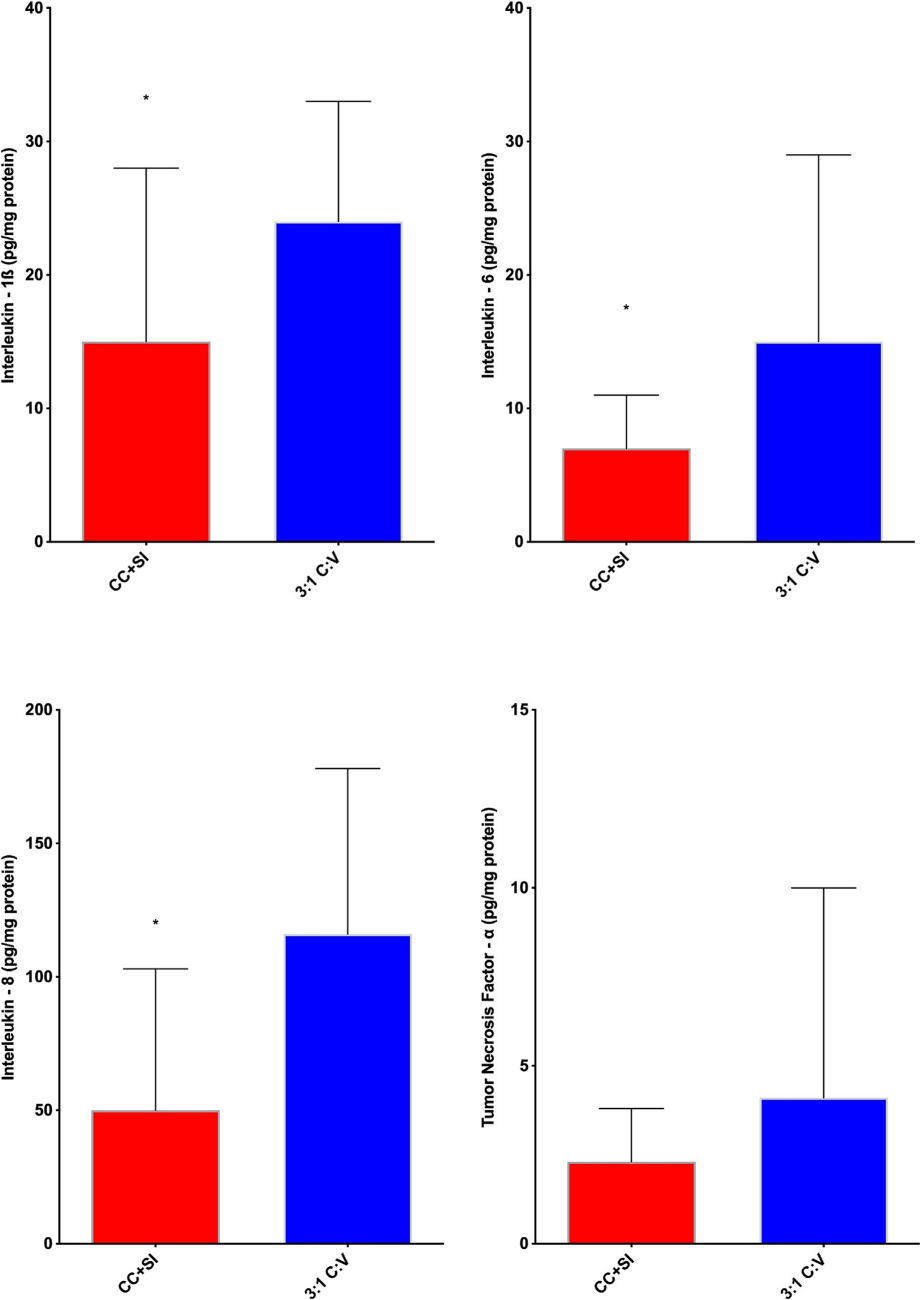
Concentrations of TNF-α, IL-1β, IL-6, and IL-8 in brain cortex tissue homogenates, expressed relative to brain cortex protein concentration in piglets resuscitated using chest compressions during sustained inflations (CC + SI) or 3:1compression:ventilation ratio (3:1C:V). Results represent the median (solid bar), IQR (box margin), and 95 % confidence interval. Asterix (*) indicated significance of p < 0.05.

There were no differences between CC + SI and 3:1C:V in the levels of injury markers IL-1ß, IL-6, IL-8, and TNF-α in the lung tissue homogenates (Fig. 5). Levels of IL-1ß, IL-6, and IL-8 were significantly lower with CC + SI compared to 3:1C:V in the brain cortex tissue homogenates (Fig. 6). Notably, none of the studies included in this analysis reported any pneumothoraxes during autopsies with CC + SI.

## Discussion

While the need for CPR at birth remains rare, infants who receive CPR have high rates of mortality and morbidities. While 3:1C:V is the current recommended approach, the approach to optimize cardiac output and oxygen delivery remains unknown. We have studied CC + SI for over a decade as a potential alternative approach during neonatal CPR and showed faster time to ROSC in piglets and human infants.^16^, ^22^, ^23^, ^33^, ^34^ Our findings can be summarized as follows: i) survival rate with CC + SI versus 3:1C:V was not different, however, ii) CC + SI shortened the required time to ROSC (Fig. 3) and improved post-resuscitation survival (Table 2), iii) mean arterial blood pressure was improved 10 min following ROSC with CC + SI, iv) minute ventilation, and therefore alveolar oxygen delivery, was significantly improved with CC + SI (Table 5), and v) injury markers (IL-1β, IL-6, and IL-8 pro-inflammatory cytokine concentrations) were lower in the brain tissue of the CC + SI group (Fig. 6). We speculate that the faster time to ROSC with CC + SI may have contributed to lower levels of inflammatory markers in the brain tissue.

### Post-ROSC survival

Providing high-quality CC to mechanically pump blood is a cornerstone to achieve ROSC, however the ventilation/inflation aspect that is combined with CC requires further examination. An often-overlooked adverse effect of CC is lung de-recruitment. Each time the chest is compressed, air is forced out of the lung, resulting in lung de-recruitment.^15^, ^35^ Compression-only resuscitation (no ventilation) in adult pigs showed that lung atelectasis significantly increased from 25 % pre-resuscitation to 75 % post-resuscitation, highlighting the impact that compressions alone have on de-recruiting the lung.^35^ During 3:1C:V, the synchronized interruption of CC to deliver an inflation led to a cumulated loss of tidal volume of 4.1 mL/kg per 3:1C:V cycle.^15^ Lung de-recruitment is concerning as it can impair oxygenation and carbon dioxide removal, resulting in delayed ROSC. In contrast, the CC + SI technique delivers a constant high distending pressure (SI) during continuous CC, which enables passive lung ventilation/aeration. A greater inspiratory than expiratory tidal volume leads to a gain in tidal volume and lung recruitment, as demonstrated by Li et al. where a gain of 2.3 mL/kg per CC + SI cycle was observed.^15^ Although air is forced out of the lung during compression of the chest, during chest recoil the high distending pressure of the SI allows gas to flow back into the lung. The result is continuous volume delivery, lung recruitment, and lung aeration. Furthermore, the CC + SI approach increases the number of inflations per minute by 4-fold, thus resulting in a significant increase in minute ventilation compared to 3:1C:V.

### Time to ROSC

The initial animal study comparing CC + SI vs 3:1C:V reported a significantly shorter mean (SD) time to ROSC with 32 (11) vs. 205 (113)sec, respectively.^16^ This was confirmed by further animal studies comparing CC + SI with 3:1C:V reporting on average a reduction in time to ROSC by ∼75 %.^16^, ^17^, ^18^, ^19^, ^20^, ^21^ These findings were also reported in two small delivery room trials, which reported that the pooled data resulted in a significant reduction in time to ROSC with CC + SI vs. 3:1C:V (mean difference 115 s, 95 % CI 184.75–45.36 sec, p = 0.001, I^2^ = 26 %).^22^, ^23^, ^24^ Similarly, in the current analysis the median (IQR) time to achieve ROSC in CC + SI piglets was significantly shorter compared to 3:1C:V piglets [90 (69–180) sec vs. 160 (77–300) sec, p = 0.0097]. Thus, both animal and human data demonstrate a shorter time to ROSC. This is reassuring as shorter time to ROSC has been associated with improved long-term neurological outcomes. However, a large trial is needed before this can be translated into clinical practice.^36^

### Injury markers

Sustained inflation as an initial ventilation strategy has been associated with increased pneumothorax rate and mortality in preterm infants <29 weeks’ gestation when compared to intermittent positive pressure ventilation.^37^, ^38^, ^39^ The exact mechanism behind the higher pneumothorax rate and mortality with sustained inflations remains unclear. Notably, none of the studies included in this analysis reported any pneumothoraxes during autopsies with CC + SI. Furthermore, a sustained inflation might deliver excessively large tidal volumes, which could trigger a pro-inflammatory pulmonary response and initiate a systemic inflammatory cascade.^40^ However, neither study using sustained inflation as an initial ventilation strategy nor any of the included studies observed an increase in lung injury markers.^41^, ^42^ Overall, CC + SI did not result in increased acute lung inflammation, as assessed by pro-inflammatory cytokine concentrations in lung tissue, compared to 3:1C:V.

Sustained inflation is thought to contribute to brain injury due to impaired venous return or excessive tidal volume delivery. Sobotka et al. reported that a single 30 sec sustained inflation followed by ventilation caused a blood–brain barrier disruption and cerebral vascular leakage, which may exacerbate brain injury in asphyxiated near-term lambs.^43^ This injury might have occurred as a direct insult of the initial sustained inflation or due to the excessive tidal volume delivered during subsequent ventilation. Shim et al. reported that a peak inflation pressure of 30 cmH_2_O during CC + SI delivered a significantly higher tidal volume compared to peak inflation pressure of 20 cmH_2_O, which was associated with significantly increased cerebral tissue pro-inflammatory cytokines.^19^ While pro-inflammatory cytokine concentrations in brain tissue had significantly lower levels of IL-1β, IL-6, and IL-8 in piglets that were resuscitated with CC + SI compared to 3:1C:V, we have not performed any other analyses to assess brain injury including staining or histology, which is a limitation of the study.

### Limitations

Our use of a piglet asphyxia model is a great strength of this translational study, as this model closely simulates delivery room events, with the gradual onset of severe asphyxia leading to bradycardia and cardiac arrest. In addition, the large number of piglets (n = 132) is a further strength of this study. However, several limitations should be considered: Our asphyxia model uses piglets that have already undergone the fetal to neonatal transition, and piglets were sedated/anesthetized. Furthermore, our model requires piglets to be intubated with a tightly sealed endotracheal tube to prevent any endotracheal tube leak; this may not occur in the delivery room as mask ventilation is frequently used. Nevertheless, our findings are still clinically relevant as the distribution of cardiac output in the fetus and post-transitional neonate during asphyxia episodes are qualitatively similar. We did not assess neurologic outcomes in surviving piglets. Furthermore, each included study had their own aim and hypothesis examining different aspects of CC + SI including lengths of the sustained inflation, peak inflation pressure, or oxygen, which could have affected the results. Parameter distributions were unbalanced across methods (e.g., FiO_2_ and rate), limiting identifiability of some interaction terms in logistic models. Pressure and duration are not defined for 3:1C:V, so their interaction terms apply within CC + SI only. Subgroup sizes (e.g., CC + SI at 90/min) were small, and interaction analyses are exploratory. Residual confounding may persist given the retrospective pooling of experiments.

## Conclusion

CC + SI reduced time to achieve ROSC and decreased brain injury markers. These studies collectively provide support for further investigation into the use of CC + SI for neonatal resuscitation.

## Funding sources

There was no funding for this work.

## CRediT authorship contribution statement

**Melanie Shaker:** Writing – review & editing, Writing – original draft, Formal analysis, Data curation, Conceptualization. **Anne Lee Solevåg:** Writing – review & editing, Validation, Methodology, Formal analysis, Data curation, Conceptualization. **Megan O’Reilly:** Writing – review & editing, Validation, Project administration, Methodology, Investigation, Formal analysis, Data curation, Conceptualization. **Georg M. Schmölzer:** Writing – review & editing, Supervision, Resources, Methodology, Investigation, Funding acquisition, Formal analysis, Data curation, Conceptualization.

## Declaration of competing interest

The authors declare that they have no known competing financial interests or personal relationships that could have appeared to influence the work reported in this paper.
